# Hypoperfusion assessed by pressure reactivity index is associated with delayed cerebral ischemia after subarachnoid hemorrhage: an observational study

**DOI:** 10.1186/s41016-021-00231-7

**Published:** 2021-03-02

**Authors:** Bin Bin Fan, Xiao Chuan Sun, Zhi Jian Huang, Xiao Min Yang, Zong Duo Guo, Zhao Hui He

**Affiliations:** Department of Neurosurgery, The First Affiliated Hospital of Chongqing Medical University, Chongqing, China

**Keywords:** Cerebral perfusion pressure, Pressure reactivity index, Delayed cerebral ischemia, Subarachnoid hemorrhage

## Abstract

**Background:**

Dysfunction of cerebral autoregulation is one of the pathophysiological mechanisms that causes delayed cerebral ischemia (DCI) after subarachnoid hemorrhage (SAH). Pressure reactivity index (PRx) have been confirmed to reflect the level of cerebral autoregulation and used to derive optimal cerebral perfusion pressure (CPPopt). The goal of this study is to explore the associations between autoregulation, CPPopt, PRx, and DCI.

**Methods:**

Continuous intracranial pressure (ICP), arterial blood pressure (ABP), and cerebral perfusion pressure (CPP) signals acquired from 61 aSAH patients were retrospectively analyzed. PRx was calculated and collected by Pneumatic computer system. The CPP at the lowest PRx was determined as the CPPopt. The duration of a hypoperfusion event (dHP) was defined as the cumulative time that the PRx was > 0.3 and the CPP was <CPPopt. The duration of CPP more than 10 mmHg below CPPopt (ΔCPPopt < − 10 mmHg) was also used to assess hypoperfusion. The percent of the time of hypoperfusion by dHP and ΔCPPopt < − 10 mmHg (%dHP and %ΔCPPopt) were compared between DCI group and control group, utilizing univariate and multivariable logistic regression. It was the clinical prognosis at 3 months after hemorrhage that was assessed with the modified Rankin Scale, and logistic regression and ROC analysis were used for predictive power for unfavorable outcomes (mRs 3–5).

**Results:**

Data from 52 patients were included in the final analysis of 61 patients. The mean %dHP in DCI was 29.23% and 10.66% in control. The mean %ΔCPPopt < − 10 mmHg was 22.28%, and 5.90% in control. The %dHP (*p* < 0.001) and the %ΔCPPopt < − 10mmHg (*p* < 0.001) was significantly longer in the DCI group. In multivariate logistic regression model, %ΔCPPopt <− 10 mmHg (*p* < 0.001) and %dHP (*p* < 0.001) were independent risk factor for predicting DCI, and %ΔCPPopt <− 10 mmHg (*p* = 0.010) and %dHP (*p* = 0.026) were independent risk factor for predicting unfavorable outcomes.

**Conclusions:**

The increase of duration of hypoperfusion events and duration of CPP below CPPopt over 10 mmHg, evaluated as time of lowered CPP, is highly indicative of DCI and unfavorable outcomes.

## Background

Cerebral autoregulation (CA) is crucial to maintain a constant cerebral blood flow (CBF) by regulating precisely brain vascular resistance in the context of changes of cerebral perfusion pressure [1]. Dysfunction of CA involves in the secondary injury after subarachnoid hemorrhage (SAH), for instance microcirculatory spasm and dysfunction contribute to cerebral ischemia as a form of secondary injury in delayed brain injury (DBI). CA can be assessed with pressure reactivity index (PRx) which has been verified in an amount of research. PRx is an autoregulatory index that calculated as a moving correlation coefficient between mean arterial blood pressure (MAP) and intracranial pressure (ICP) [2]. PRx has values in a range between − 1 and + 1, a negative or 0 value reflects a physiological CA, but positive values reflect dysfunction of CA [2] [3]. Optimal cerebral perfusion pressure (CPPopt) can be calculated by PRx, which develops into cerebral perfusion pressure (CPP) treatment protocols, patients with CPP close to their calculated optimum had better outcomes than those with large deviations, proven effectiveness in traumatic brain injury (TBI) [4–6] .

Delayed cerebral ischemia (DCI) is defined as a clinical deterioration along with associated ischemic changes and hypoperfusion [7]. DCI is an important predictor of mortality and morbidity in SAH patients [8]. The mechanism of dysfunction of CA to cause DCI is definite, and a series of findings suggest that measurement of CA can be used for early identification DCI [9–11]. Due to the lacking method of continuous monitoring for CA in SAH patients, clinical evaluation of CA in patients is mostly done by static method such as Xe-CT scan, MRI, and PET [12].

In our study, we assessed the state of cerebrovascular reactivity and CPPopt in SAH patients, and exploratively investigate the relationship between hypoperfusion identified by PRx threshold and DCI.

## Methods

### Patient population

Aneurysm subarachnoid hemorrhage (aSAH) patients were diagnosed with intracranial aneurysm by DSA or CTA, and treated by microsurgical clipping and intracranial pressure (ICP) monitoring. Traumatic SAH patients or other cerebrovascular diseases were excluded. Arterial blood pressure (ABP) and ICP obtained in 61 patients were collected at the neuro-intensive care unit (NICU) at the First Affiliated Hospital of Chongqing Medical University between January 2018 and September 2019. All data were analyzed retrospectively. A standardized treatment protocol was used, focusing on early detection and treatment of secondary insults [13]. Propofol and morphine were used for sedation and analgesia, respectively. ICP targets were < 20 mmHg and CPP > 60 mmHg. Other aims of the treatment protocol were normotension, normovolemia, body temperature < 38 °C, and normal electrolyte levels.

### Definition of DCI

All patients with a diagnosis of aSAH were strictly followed by a standardized imaging protocol in all cases. Brain CT scan, CTA, and a CT perfusion study were performed on admission and/or prior to transfer to the First Affiliated Hospital of Chongqing Medical University. Patients after microsurgical clipping were admitted and managed in NICU. Brain CT scan were performed on the first day after surgery and before being transferred out of the NICU in all patients. The determination of performing additional CT in NICU depended on level of consciousness in patients and associated complications. Brain MR scan were performed in all patients before discharge.

Diagnosis of DCI was based on standard definition including clinical deterioration and cerebral infarction [8]: clinical deterioration caused by DCI was defined as the occurrence of a new focal neurological impairment such as hemiparesis, aphasia, apraxia or hemianopia, or a deterioration of consciousness by two points on the Glasgow Coma Scale that is sustained for at least 1 h. These symptoms cannot be apparent immediately after aneurysm treatment. Some factors that could lead to similar clinical findings, such as postoperative hematoma, iatrogenic ischemia and/or infarction, edema, hydrocephalus, metabolic derangement, seizures, and infection were excluded. Cerebral infarction was defined as the presence of cerebral infarction on CT or MR scan of the brain within 6 weeks after SAH. Cerebral ischemia present on CT or MR between 24 and 48 h after aneurysm occlusion, and cerebral ischemia attributable to other causes such as surgical clipping should be excluded. The diagnosis of DCI was independently diagnosed by 2 or more neurosurgeons.

### Data acquisition and processing

ICP was monitored with an intraparenchymal probe (Sophysa ICP Micro-Sensor, BJM428, France). ABP was monitored through the radial artery with the aid of a standard pressure monitoring kit and was zeroed at the level of the Monro. Data were sampled at 100 Hz with Pneumatic computer for proprietary data acquisition and analysis [14]. ABP and ICP signals were first averaged (mean) over a 10-s window. The PRx was calculated as a short-term moving Pearson correlation coefficient between changes in 30 consecutive 10-s averages of ABP and corresponding ICP.

### Estimation of CPPopt

**Fig. 1:**
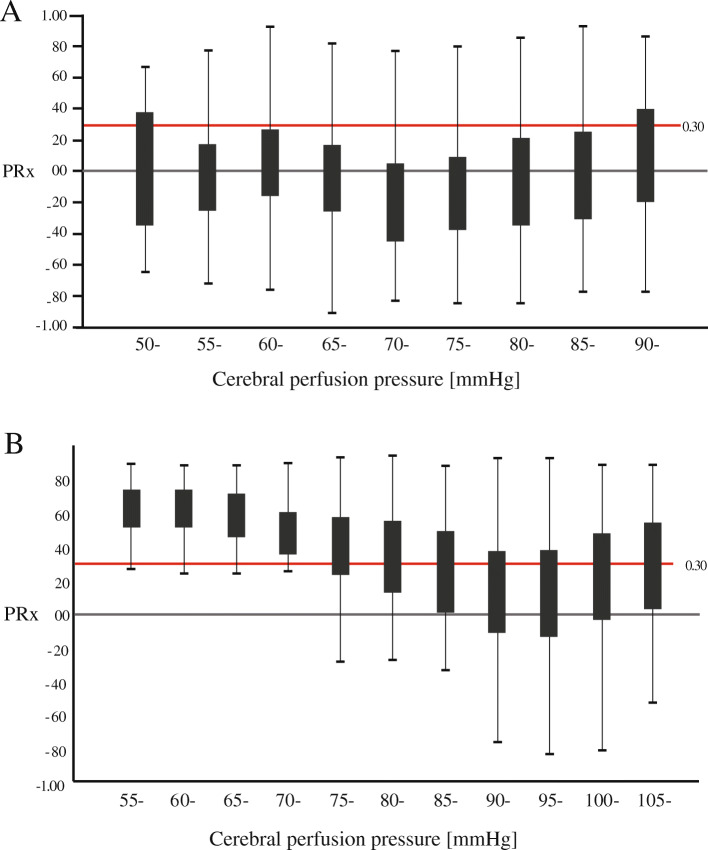
Assessment of CPPopt in two exemplary patients. The pressure reactivity index (PRx) is plotted against cerebral perfusion pressure (CPP). ‘U-shaped’ curve relationships between CPP and PRx including estimation of optimal CPP (CPPopt) is obtained. PRx values > 0.3 indicate impaired pressure reactivity. The patient A reaches the minimum PRx for CPP values around 70–75 mmHg. The patient B has the CPPopt around 95–100 mmHg

CPP (CPP = ABP − ICP) were calculated using waveform time integration over 10-s intervals. CPP-PRx curve was calculated with the method proposed by Steiner [4]. CPP values were divided into groups of 5 mmHg and PRx was averaged within these groups. These data were plotted as a curve with CPP groups on the *x*-axis and mean of PRx on the *y*-axis in all patients. The CPPopt were defined as CPP associated with the lowest values of PRx in “U-shaped curve” (Fig. 1). For obtaining accurately CPPopt in all patients, we used all data during the period of monitoring to draw CPPopt curve instead of U-shaped curve fitting from a period of several hours using fitting software [15].

### Assessment of hypoperfusion by PRx

Previous studies have found that both low and high CPP resulted decline of cerebral vascular reactivity, so that threshold method was used to identify poor CPP. A similar index by Hakseung Kim [16] which was named the duration of a hypoperfusion event (dHP) was used to assess hypoperfusion. The dHP was defined as the cumulative time that the PRx was > 0.3 and the CPP was < CPPopt. The value of 0.3 as the threshold for PRx to identify poor CPP was chosen as it had been identified determining fatal outcome in TBI patients [17]. Another index was the duration of CPP more than 10 mmHg below CPPopt (ΔCPPopt < − 10 mmHg) [18]. Both two indices were calculated in those acquiring CPPopt patients.

### Statistical analysis

dHP and time with ΔCPPopt < − 10mmHg was compared between DCI group and control group, utilizing univariate and multivariable logistic regression. Pearson’s chi-squared test and continuity-adjusted chi-squared were used for comparisons of the rate in four-fold table data. *t* test and rank sum test were used to analyze the differences measurement data between groups. Univariate and multivariable logistic regression were used to analyze in the end. According to modified Rankin score (mRs) at 3 month**s**, and logistic regression and ROC analysis were used for predictive power for unfavorable outcomes (mRs 3–5). Age (years), Hunt-Hess Score, mFisher Score [19], and dHP or ΔCPPopt < − 10 mmHg were included as Predictors. We used the Statistical Product and Service Solutions (SPSS) (SPSS version 26.0) for data analysis. The significance level was set at 0.05.

## Results

### Patient characteristics (Table 1)

In a total over 3300 h, median total monitoring time was 52 h (IQR 64, range13 to 133 h) continuous bedside monitoring was performed on 61 patients (24males and 37 females) at NICU at the First Affiliated Hospital of Chongqing Medical University between January 2018 and September 2019. The mean initial time of monitoring was 4 days after aneurysm rupture. The mean age was 53.57 years, median CT Fisher score was 2 (range 1–4), and median Hunt-Hess grade was 3 (range 1–4). Four patients were excluded by reason of decompressive craniectomy. CPPopt could not be calculated in 5 patients. Data from the 52 patients were used for further analysis finally (Table 1). The prognosis was evaluated at 3 months after discharge. Forty-four patients (85%) had a good recovery (mRs 0–2), and mortality was 0. Table 1 showed no significant difference on age, sex and aneurysm location between DCI group and control group. The poor outcomes (mRs range 3–5) in DCI was significantly higher than control group (35% vs 6%, *p* = 0.009).

**Table 1:** Characteristics of 52 patients

Characteristic	Control *n* = 35 (%)	DCI *n* = 17 (%)	*p*
Age [years] (mean (sd))	52.00 (10.25)	55.59 (8.47)	NS
Female sex	20(57)	10(59)	NS
Aneurysm location			NS
ACoA	7(20)	3(18)	NS
ICA	9(26)	5(29)	NS
MCA	19(54)	9(53)	NS
Fisher grade			
1	22(63)	7(41)	
2	9(26)	0	
3	4(11)	8(47)	
4	0	2(12)	
Hydrocephalus	3(9)	5(29)	NS
mRs (3–5)	2(6)	6(35)	0.009

*NS* = No significant

### Physiological description (Table 2)

The mean ICP was significantly higher in those that DCI (12.43 ± 5.13 mmHg, *p* = 0.002). The monitoring time in NICU was significantly longer in those that DCI (71.94 ± 18.43 h, *p* = 0.003). The mean PRx was suggestive for impaired pressure reactivity defined as PRx > 0.3 in 9 patients (17%), but there was no significant difference between DCI and control (0.23 ± 0.10 vs 0.19 ± 0.09, *p* = 0.061). Figure 1 demonstrated two exemplary patients to depict the CPPopt concept. The CPPopt was calculated in 52 patients (91%). The mean CPPopt was significantly higher in those that DCI (77.35 ± 12.39 mmHg, *p* = 0.006).

**Table 2:** physiological description of the cohort

Variable	Control	DCI	*p*
*n*	35	17	
Monitoring time[h] (mean (sd))	47.31(9.36)	71.94(18.43)	0.003
ABP [mmHg] (mean (sd))	87.15(8.35)	89.28(8.82)	0.201
ICP [mmHg] (mean (sd))	8.01(3.25)	12.43(5.13)	0.002
CPP [mmHg] (mean (sd))	79.14(8.22)	76.85(10.69)	0.199
PRx (mean (sd))	0.19(0.09)	0.23(0.10)	0.061
CPPopt [mmHg] (mean (sd)	68.57(6.25)	77.35(12.39)	0.006
%dHP (mean (sd))	10.66(11.44)	29.23(18.52)	< 0.001
%ΔCPPopt <− 10 mmHg (mean (sd))	5.90(7.38)	22.28(15.10)	< 0.001

*ICP* intracranial pressure, *CPP* cerebral perfusion pressure, *PRx* pressure reactivity index, - optimal cerebral perfusion pressure, *ΔCPPopt* CPP-CPPopt

### Description of hypoperfusion (Table 2 and Fig. 2)

We calculated separately percent of the time of hypoperfusion by dHP and ΔCPPopt < − 10 mmHg in each patient. Hypoperfusion events were found in every single patient. The mean % dHP in DCI was 29.23%, and 10.66% in control. The mean %ΔCPPopt < − 10 mmHg was 22.28% and 5.90% in control. The time of hypoperfusion defined as dHP (*p* < 0.001) and ΔCPPopt < − 10 mmHg (*p* < 0.001) was significantly longer in the DCI group.

**Fig. 2:**
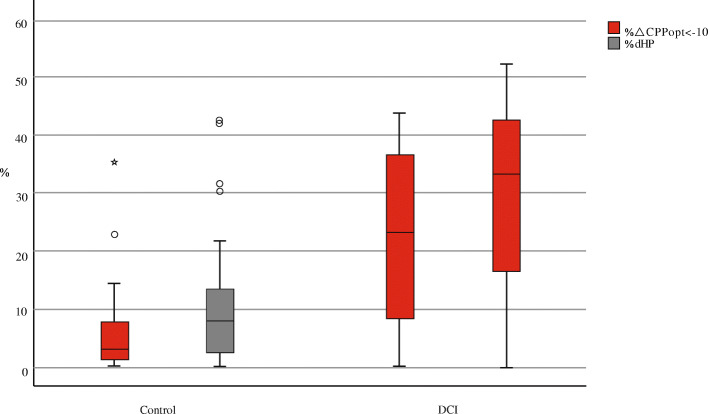
Comparison of hypoperfusion between control and DCI. The time of hypoperfusion defined as %dHP and %ΔCPPopt < − 10 mmHg was significantly longer in the DCI group

### Multivariable logistic regression model and ROC analysis (Table 3 and Fig. 3)

In univariate logistic regression, ICP (*p* = 0.004), mFisher Score (*p* = 0.006), Hunt-Hess Score (*p* = 0.020), %ΔCPPopt <− 10 mmHg (*p* < 0.001) and % dHP (*p* < 0.001) were significantly associated with DCI. In the covariate-adjusted logistic regression model, %ΔCPPopt <− 10 mmHg (*p* = 0.006) and % dHP (*p* = 0.047) were significantly associated with DCI. The odds ratio (OR) for dHp% was 1.055 (95% CI 1.001–1.112); the OR for ΔCPPopt% was 1.114 (95% CI 1.030–1.204). Furthermore, %ΔCPPopt <− 10 mmHg (0.909, 95% CI 0.825–0.993) was found to have the highest AUC over % dHP (0.876, 95% CI 0.770–0.0.981), indicating that it had better discriminative ability.

**Table 3:** Logistic regression model for indices of hypoperfusion and ROC analysis of predictive power for DCI in patients with aSAH

	Univariate *p* value	AUC	95% CI	Covariate-adjusted *p* value	AUC	95% CI
%dHP	< 0.001	0.761	0.593–0.928	0.047	0.876	0.770–0.981
%ΔCPPopt < − 10 mmHg	< 0.001	0.810	0.660–0.961	0.006	0.909	0.825–0.993

Adjusted variables including ICP, Hunt-Hess Score, and mFisher Score

**Fig. 3:**
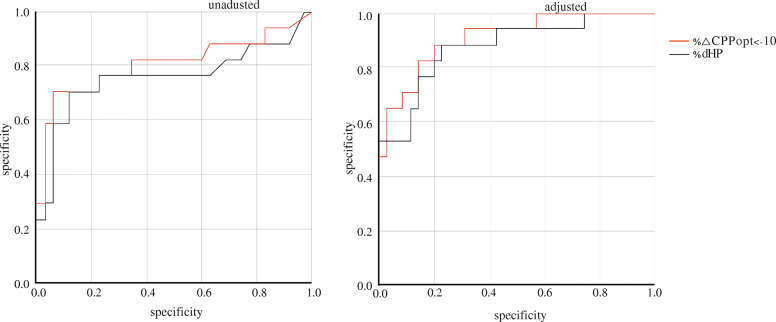
Comparison of receiver operator characteristic (ROC) curves for predicting DCI using logistic regression model. Both unadjusted and adjusted for covariates. The larger area under the ROC curve indicates superiority of %ΔCPPopt < − 10 mmHg over %dHP; also, inclusion of covariates improves the discriminative ability of the markers. Adjusted variables including ICP, Hunt-Hess Score, and mFisher Score

### Multivariable logistic regression model to predict unfavorable outcomes (Table 4)

In the covariate-adjusted logistic regression model, %ΔCPPopt <− 10 mmHg (*p* = 0.010) and % dHP (*p* = 0.026) were significantly associated with unfavorable outcomes (mRs 3–5). The OR for dHp% was 1.091 (95% CI 1.010–1.178); the OR for ΔCPPopt% was 1.133 (95% CI 1.034–1.242). Unfavorable outcomes at 3 months was predicted by %ΔCPPopt < − 10 mmHg (0.955, 95% CI 0.877–1.000) or % dHP (0.923, 95% CI 0.819–1.000). Age (years), Hunt-Hess Score, and mFisher Score were included as adjusted variables.

**Table 4:** Logistic regression model for indices of hypoperfusion and ROC analysis of predictive power for unfavorable outcomes (mRs 3–5) in patients with aSAH

	Univariate *p* value	AUC	95% CI	Covariate-adjusted *p* value	AUC	95% CI
%dHP	0.005	0.838	0.707–0.969	0.026	0.923	0.819–1.000
%ΔCPPopt < − 10 mmHg	0.008	0.864	0.724–1.000	0.010	0.955	0.877–1.000

Adjusted variables including age, Hunt-Hess Score, and mFisher Score

## Discussion

In this study of patients with aSAH, we had used the method of continuous monitoring of cerebrovascular reactivity by PRx to explore how to identify hypoperfusion events and estimated the CPPopt. We demonstrated two indices—dHP and ΔCPPopt < − 10 mmHg were associated with DCI. The two indices were verified by multivariate analysis and the logistic regression model was established for predicting DCI and unfavorable outcomes.

### Dysfunction of CA and DCI in SAH

In the past, cerebral vasospasm had been recognized as a major cause of DCI, but it has been questioned. Vasospasm has been successfully reduced in some studies, but in fact the outcome was not improved [20]. Pathophysiological causes of DCI include microthrombic, microvascular constriction, inflammation, cortical spreading ischemia, and blood–brain barrier disruption; vasospasm is a cause of vascular constriction. That is why vasospasm was only observed in part of patients with DCI. On the contrary, patients with vasospasm do not necessarily cause DCI. Development of DCI, relate to the automatic regulation of the brain, depends on the extent and severity of vasospasm, as well as on existing collateral and anastomotic blood flow, cerebral metabolic demand, blood pressure, and other such parameters [21]. In our research, patients with dysfunction of CA were most in DCI group.

### PRx reflects automatic regulation

**Fig. 4:**
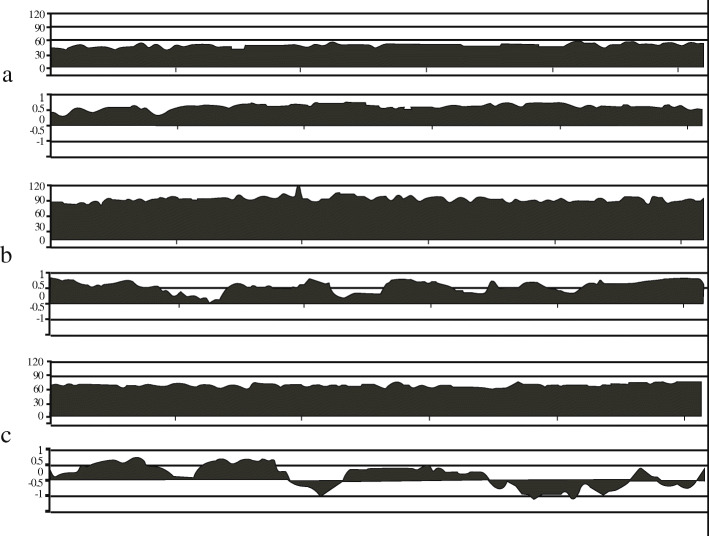
Example of PRx reflecting cerebrovascular reactivity at different levels of CPP (in a 20-min period). **a** At the mean CPP was 55 mmHg, PRx was around 0.5 abidingly. **b** At the mean CPP was 90 mmHg level, PRx was greater than 0 continuously and the mean PRx value was more over 0.5. **c** At the mean CPP was CPPopt (65–70 mmHg), the phenomenon of random fluctuation of PRx at 0 was observed. It reveals difference of cerebrovascular reactivity at varied CPP

The relationships between CPP and PRx have been emphasized as ‘U-shaped’ curve. When CPP remains range within autoregulatory plateau, PRx value is a negative or 0 value, and it reflects a good cerebrovascular reactivity. Positive values reflect dysfunction of CA during CPP is out of the autoregulatory plateau [18]. CA depends on cerebrovascular resistance gradient. Resistive arterioles like pial arteriole and large, and conduit arteries like internal carotid arteries conjointly regulate CA [22] [23]. The variational volume of cerebral arteries by the changing of CPP results in an increasing or decreasing ICP, and PRx reflects the signal feature of it. The degree of random of PRx is reducing when CPP approaches the limits of pressure reactivity far from autoregulatory plateau. In other words, cerebrovascular reactivity reservation has been lost, and the CA function has failed, as shown in Fig. 4. It can be explained that a physiological process occurs presumably, mainly regulator of cerebral arteries system reactivity become pressure regulation instead of brain metabolism regulation. In our cohort, mean PRx over 0.3 was found in 9 patients (17%). In a single patient, time with PRx over 0.3 existed for a while during the period of monitoring, whereas there was no significance between DCI and control. It is due to that hypoperfusion and hyperperfusion lead to increasing PRx value, yet the pathological mechanism of DCI is mainly related to hypoperfusion.

### Indexes of hypoperfusion by PRx and CPP

The mean CPPopt was significantly higher in DCI group, the mean CPP of the two groups were almost equal. It is similar with report by Philippe Bijlenga [24]. It reveals the phenomenon of a CPPopt rightward shifted caused by dysfunction of autoregulation. It is an important one of the complex mechanisms of DCI. Factors such as hypercapnia or hypocapnia [12], or the microvascular spasm will probably result in a shift of the autoregulatory plateau to the right and eventually lead to DCI [25]. The mean CPP in our cohort was over 60 mmHg; however, hypoperfusion event had occurred. It suggest that CPPopt may be a better strategy to manage CPP instead of single thresholds of CPP. Similar pieces of evidence about flexible CPP thresholds by PRx like CPPopt have been proven effective in recent studies [18] [26] [27].

Clinical methods for monitoring cerebral blood flow including CTP, MR, and TCD have some limitations. One of limitations is brain perfusion can only be monitored for a certain period of time instead of the whole process. We combined PRx and CPPopt to identify hypoperfusion events. The dHP and ΔCPPopt < − 10 mmHg can effectively identify hypoperfusion and its outcome events. It has similar with research by Hakseung Kim in TBI [16]. In the logistic regression model, we found that dHP and ΔCPPopt < − 10 mmHg were associated with unfavorable outcomes. It may be that the DCI caused by hypoperfusion leads to permanent neurological dysfunction in aSAH patients. In our study, we found that as the Hunt-Hess scores, so do %ΔCPPopt < − 10 mmHg and %dHP, there was not statistically significance. It can be explained with that CBF disturbances related to the severity of SAH [28]. End events about cerebral luxury perfusion were not observed in the study, and it is ascribed to that more efficacious buffering capacity against increases than decreases in perfusion pressure of CA [29].

## Limitations

This study has several important limitations. Retrospective studies are susceptible to bias, despite the fact that some of the inherent limitations of a retrospective study are overcome due to we collected data prospectively. And by nature of its observational design, conclusions about whether CPPopt protocol will improve patient physiology or outcome are unforeseeable. Nevertheless, the extension of the method described here seems crucial for the design of different prospective trials. Practical and safety issues might guide choices between strict flexible targets, flexible thresholds, or even flexible ranges.

Calcium antagonists (Nimodipine) are routinely used to improve the outcome in aSAH [30]. Calcium antagonists have a direct effect on vascular smooth muscle cells and alter CA, and it has been verified in humans [31] [32]. Although there was a study on SAH patients found that Nimodipine affected one autoregulatory index (ORx) but not PRx [33]. It is difficult to assess the continuous influence of Nimodipine on PRx and CA in the study.

Finally, our study with a small sample size; therefore, we have some caveats regarding significance testing. The results must be interpreted critically. For example, there is also an increased risk of significant findings being false positives, and we can decrease these risks by increasing the sample size. Practically, the present study is to be considered the generation of a hypothesis rather than hypothesis testing and needs more researches to test.

## Conclusions

Our study demonstrates that PRx can assess cerebrovascular reactivity and be used to calculate CPPopt in aSAH patients. The increase of duration of hypoperfusion events and duration of CPP below CPPopt over 10 mmHg, evaluated as time of lowered CPP, is highly indicative of DCI and unfavorable outcomes.

## Data Availability

Not applicable.

## Abbreviations

CA: Cerebral autoregulation
CBF: Cerebral blood flow
SAH: Subarachnoid hemorrhage
PRx: Pressure reactivity index
ICP: Intracranial pressure
ABP: Arterial blood pressure
CPP: Cerebral perfusion pressure
CPPopt: Optimal cerebral perfusion pressure
dHP: Duration of hypoperfusion
